# *Ruellia tuberosa L.* Extract Improves Histopathology and Lowers Malondialdehyde Levels and TNF Alpha Expression in the Kidney of Streptozotocin-Induced Diabetic Rats

**DOI:** 10.1155/2020/8812758

**Published:** 2020-10-14

**Authors:** Anna Roosdiana, Fajar Shodiq Permata, Riera Indah Fitriani, Khairul Umam, Anna Safitri

**Affiliations:** ^1^Chemistry Department, Brawijaya University, Jl. Veteran, Malang, 65145, Indonesia; ^2^Faculty of Veterinary Medicine, Brawijaya University, Puncak Dieng Ekslusif, Malang, 65151, Indonesia; ^3^Research Center for Smart Molecules of Natural Genetic Resources (SMONAGENES), Brawijaya University, Jl. Veteran, Malang, 65145, Indonesia

## Abstract

*Ruellia tuberosa L.* is a therapeutic plant that is generally consumed in Indonesian traditional medicine to prevent or cure various illnesses, i.e., diabetes. The current study was conducted to investigate the effects of hydroethanolic root extracts of *Ruellia tuberosa L.* on the kidney of streptozotocin-induced diabetic Wistar rats. In this study, male Wistar rats were divided into 5 groups: healthy rats (group 1), diabetic rats (group 2), and treated rats which received extract at dosages of 250 (group 3), 375 (group 4), and 500 (group 5) mg/kg body weight for 21 days. Diabetes mellitus was experimentally induced by the administration of five doses of streptozotocin 20 mg/kg body weight within five consecutive days. Significant increases in the value of TNF alpha expression and malondialdehyde (MDA) levels were observed in streptozotocin-induced diabetes rats. Furthermore, severe histological alterations of kidney tissues occurred in the diabetic rats group. After treatment was applied, the value of TNF alpha expression and MDA levels on the kidney decreased considerably (*p* < 0.05) in groups 3, 4, and 5. The optimum dosage was obtained at a dose of 250 mg/kg body weight (group 3), which had 42.24% and 52.70% decrease in TNF alpha expression and MDA levels, respectively. The histopathological profiles of the kidney also showed significant improvements in treated groups. The most prominent recoveries were also shown in group 3. The treatments induced repairment in the glomerular and renal tubular damages in the kidney tissues. To conclude, these results emphasize potentially health valuable properties of hydroethanolic root extracts of *R. tuberosa L*. in rats with streptozotocin-induced diabetes.

## 1. Introduction

Diabetes mellitus (DM) is a group of metabolic disorders described by hyperglycemia that occurs due to defects such as insulin secretion, decreased sensitivity of insulin receptors, or both. This disease is considered as a serious health problem in the world because it is predicted that the number of people with DM will continue to increase each year [1]. Generally, there are two types of DM, namely, DM type 1, or referred to as insulin-dependent DM, and DM type 2, or referred to as noninsulin-dependent DM/NIDDM [2].

Diabetic nephropathy (DN) is one of the acute DM complications. In patients with DN, kidney histopathological damage is indicated by thickening of the glomerular basement membrane, expansion of mesangial cells, macrophage infiltration, damage of podocytes, and tubular epithelium disintegration [3, 4]. In recent years, oxidative stress is suggested as the major cause contributing to DN. The pathogenesis of DN can be caused by decreasing antioxidants action and increasing production of prooxidants [5]. In the diabetic state, hyperglycemia was suspected to generate inflammation, oxidative stress, apoptosis, and kidney fibrosis. Moreover, hyperglycemia was reported to accelerate the formation of advanced glycation end (AGE) and its receptor RAGE [6]. Besides, in DN, protein leak into the urine is a result of glomerular damages that linked to raised levels of creatinine and blood urea [7]. The development of DN eventually will result in the final step of renal failure [8].

Malondialdehyde (MDA) is a compound consisting of three carbons that resulted from polyunsaturated fatty acids peroxidation, i.e., arachidonic acid. It is one of the final products of membrane lipid peroxidation. Since MDA levels are elevated in numerous diseases with an excess of oxygen-free radicals, MDA has been associated with free radical damages [9, 10]. The higher the damages caused by oxidative stress, the higher the levels of MDA.

Diabetes mellitus in animal models can be created by inducing streptozotocin (STZ) as a diabetogenic agent [11]. Streptozotocin is a donor of nitric oxide (NO) that contributes to cell damage. Damages in pancreatic beta cells begin when STZ penetrates Langerhans beta cells through the GLUT-2 glucose transporter which results in DNA alkylation of pancreatic beta cells [12]. Moreover, NO in mitochondria causes an increase in xanthine oxidase activity that catalyzes the formation of superoxide anion; in the presence of hydrogen peroxide, superoxide anion and superoxide radicals are formed, which also cause DNA damage. This can activate the ADP-poly-ribosylation which then results in suppression of NAD^+^ and decreases in the amount of ATP and eventually inhibition of insulin secretion and synthesis [13].

Treatment of diabetes mellitus is generally conducted by insulin injection and the consumption of antidiabetic oral drugs [1]. However, these methods require large costs and are at risk of causing unsafe side effects. The high cost of DM treatments initiates clinician and researchers to search for alternative medicines from natural ingredients that are affordable and has minimal side effects compared to treatment using chemical drugs.

*Ruellia tuberosa L*. is one of the medicinal plants that is widely distributed in tropical countries, i.e., Indonesia. In herbal medicine, this plant has been widely used as an antidiuretic, antidiabetic, analgesic, and antihypertensive [14, 15]. Previously, *n*-hexane root extracts of *R. tuberosa L*. had shown antidiabetic activity through *in vivo* studies [16, 17]. These effects included decreasing blood glucose concentrations, lowering levels of MDA, and showing improvements on the kidney histopathological profiles [16, 17]. Phytochemical examination of this study showed that *n*-hexane *R. tuberosa L*. root extracts containing triterpenoid compounds [17]. Furthermore, we have conducted an *in vivo* study on *R. tuberosa L*. hydroethanolic root extracts and found that the extracts lowered blood glucose levels and MDA levels on serum [18] and MDA levels on the pancreas [19] and also had effects on serum enzyme activity [20]. Phytochemical screening tests conducted on this study discovered that *R. tuberosa L.* roots extracted with ethanol and water containing mostly phytosterol and flavonoid compounds [21, 22]. The hydroethanolic root extracts of *R. tuberosa L*. biological and pharmacological activities on the kidney of diabetic Wistar rats have not been previously reported. In this work, we report the effects of the hydroethanolic root extracts of *R. tuberosa L*, on the histopathology profiles, MDA concentrations, and TNF alpha expression of the kidney.

## 2. Materials and Methods

### 2.1. Animals and Experimental Design

Preparation of the hydroethanolic extracts was previously reported as in [23]. Preparations of animal models and all treatments applied were the same as in our previous studies [19]. In brief, 20 male Wistar rats (body weights 120–180 g) were acquired from the Biosains Institute, Brawijaya University. This study was accepted by the Ethics Committee of Brawijaya University (approval no. 873-KEP-UB). Upon arrival, animals were acclimatized in individual caged rooms for 1 week with controlled temperature (22 ± 3°C) and lighting cycle of 12 h light and 12 h dark. All experimental animals were freely given tap water and standard feed. After adaptation, rats were divided into five groups (*n* = 5 in each group): control (group 1); diabetic (group 2); diabetic that treated with 250 mg/kg body weight extracts per day (group 3); diabetic that treated with 375 mg/kg body weight extracts per day (group 4); and diabetic that treated with 500 mg/kg body weight extracts per day (group 5). The extract doses were chosen based on the doses applied to our previous study [17]. Diabetes mellitus was induced by STZ injection intraperitoneally with 20 mg/kg body weight in 100 mL of citrate buffer for five days consecutively. The STZ was given as multiple low dose based on the previously published study [11]. Diabetes was checked by measuring glucose levels in blood samples obtained from the tail of the animals with a glucometer. The rats in groups 3, 4, and 5 received treatments for 21 days, starting one week after STZ injection. All the treatments were carried out by oral gavage and stopped after three weeks.

### 2.2. Histopathological Examination and Immunohistochemistry

Rats were euthanized, and the kidney was removed from every rat, weighed, and washed with ice-cold normal saline. The collected kidney samples were fixed in 10% neutral-buffered formaldehyde for 4 h and embedded in paraffin. The paraffin blocks were cut into 4 *μ*m sections and stained with haematoxylin and eosin for histopathological examinations. The other sections were placed in PBS pH 7.4 for 15 min and then to block endogenous peroxidase activity treated with 3% H_2_O_2_ for 10 min. After that, the sections were washed with PBS pH 7.4 and repeated 3 times at room temperature. Then, these were induced with primary antibody (anti-rat TNF alpha) for 24 h, at 4°C, washed again with PBS pH 7.4, incubated with secondary antibodies for 1 h at room temperature, and then washed again using PBS pH 7.4. Furthermore, the kidneys that have been stained with immunohistochemical staining were observed under a microscope using 400× magnification processed using ImmunoRatio software.

### 2.3. Measurement of MDA Levels

The determination of MDA levels was carried out using spectrophotometry UV-Vis technique, with TBA reagents. The kidneys' rats from all groups (1 to 5) were cut into small pieces and crushed. Then, kidney physiological 0.9% NaCl was added to kidney homogenates and centrifuged for 20 min at a speed of 8000 rpm. The supernatants were collected and diluted with distilled water and TCA and then homogenized with vortex. The HCl 1 N was added to the mixture, mixed with 1% sodium thiosulphate solution, and then homogenized with a vortex. After that, the mixture was centrifuged at a speed of 500 rpm for 15 min. The supernatant was incubated at a water bath at 100°C for 30 min and cooled at room temperature. The color changes on the samples after adding of TBA reagent were measured using a UV-Vis spectrophotometer (Shimadzu UV-visible spectrophotometer UV-1601) at the 530 nm wavelength. The absorbance values were proportional to the concentration of MDA in the samples.

### 2.4. Statistical Analysis

Statistical analyses were conducted using Statistical Package for The Social Science (SPSS) version 23.0 for windows. The data will be expressed as mean ± SEM. The one-way analysis of variance (ANOVA) followed by Tukey's post hoc test was used for the analysis of data. Results are written as mean ± SEM. The differences at *p* < 0.05 were considered statistically significant. Histopathological features of the kidney were analyzed qualitatively.

## 3. Results

The effect of therapeutic extracts of hydroethanolic root extracts of *R. tuberosa L* can be seen from changes in MDA levels. The results of MDA level measurements are shown in Table 1. In the diabetic group (group 2), MDA levels increased up to 281%. After treatment with *R. tuberosa L* extracts, MDA levels in treated groups (groups 3, 4, and 5) decrease significantly at *p* < 0.05. The lowest dose used (250 mg/kg body weight) resulted in the highest decrease by 52.70%. In contrast, the highest dose at a dose of 500 mg/kg body weight had MDA levels decreased by 30.15%, which was the lowest decrease among the treatments.

**Figure 1:**
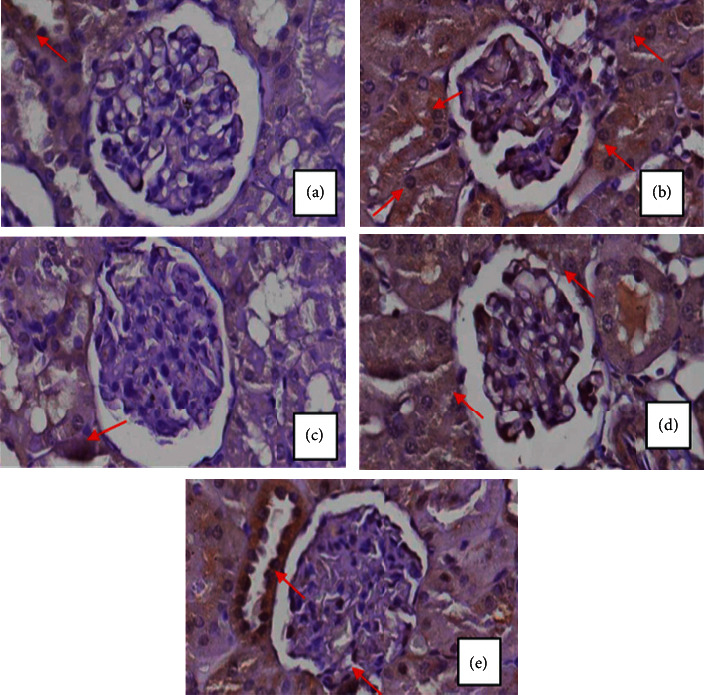
TNF alpha expression in the kidney of (a) healthy rats (group 1), (b) diabetic rats (group 2), (c) diabetic rats that treated with 250 mg/kg body weight extracts per day (group 3), (d) diabetic rats that treated with 375 mg/kg body weight extracts per day (group 4), and (e) diabetic rats that treated with 500 mg/kg body weight extracts per day (group 5). The red arrows show renal tubules with TNF alpha expression.

Analysis of TNF alpha expression was performed by immunohistochemical staining; these were indicated by the presence of a brownish color, as shown in Figure 1 with brownish color arising from the bond between TNF alpha, TNF alpha anti-rat secondary antibody, IgG anti-rat, SA horseradish peroxidase, and DAB chromogen. Decreased expression of TNF alpha in the kidneys is shown by the decreasing brownish color in the tubules (Figure 1)(b). After treatments were applied, TNF alpha expression increases, as shown in Figures 1(c) and 1(d). The quantification of TNF alpha expression was carried out in as many as 5 visual fields using the ImmunoRatio software application. Each treatment group was analyzed with SPSS software using the One-Way ANOVA test and with a level of confidence *α* = 5% (Table 2).

From Table 2, it can be seen that group 1 had the lowest TNF alpha expression value (18.78). TNF alpha expression in the diabetic group (group 2) was drastically increased (185%), following streptozotocin induction. Increase in the TNF alpha expression is a response to the high tissue inflammation that occurred in the diabetic states. After treatments were applied, the TNF alpha expression was lowered significantly. Treatment with a dose of 250 mg/kg body weight resulted in the highest decrease (42.24%) of the TNF alpha expression. This was followed by doses of 375 and 500 mg/kg body weight, with 35.51% and 20.12% decrease in TNF alpha expression, respectively. These results were in agreement with those in the trend on the doses of the therapy to the MDA levels. The higher therapy dose indicates the lower decrease in TNF alpha expression.

**Figure 2:**
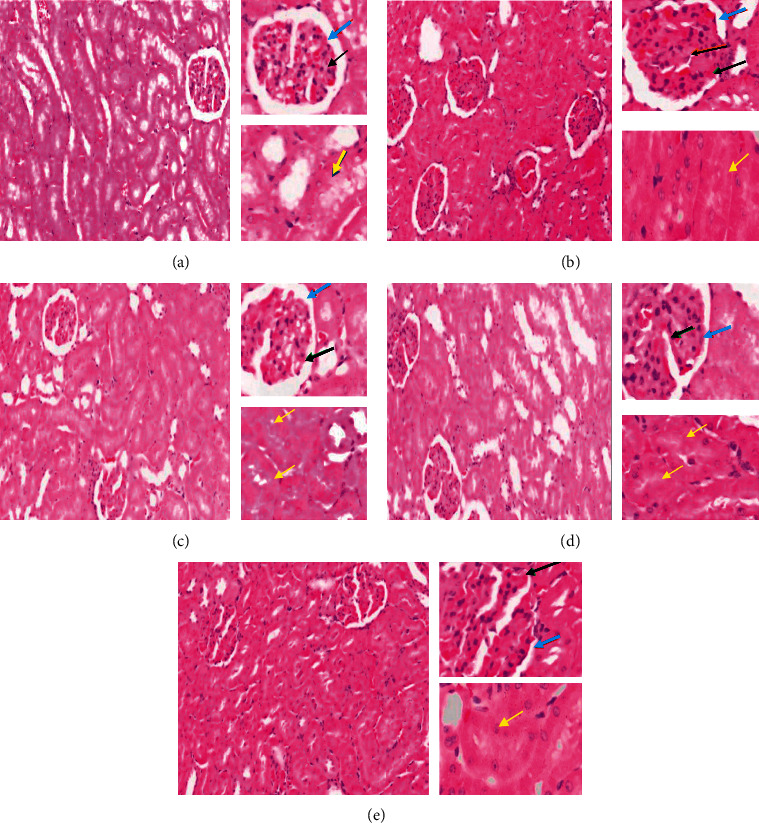
Histopathological profiles of the kidney of (a) healthy rats (group 1), (b) diabetic rats (group 2), (c) diabetic rats that treated with 250 mg/kg body weight extracts per day (group 3), (d) diabetic rats that treated with 375 mg/kg body weight extracts per day (group 4), and (e) diabetic rats that treated with 500 mg/kg body weight extracts per day (group 5). Number 1 shows overview of the kidney with 10× magnification, number 2 shows glomerulus area with 40× magnification, and number 3 shows tubules with 40× magnification. Black arrows show glomerulus, yellow arrows show proximal tubules, and blue arrows show Bowman's space.

The histopathology of the renal glomerulus in the healthy rats group (Figure 2(a)) shows an image of normal kidney tissues, with the shapes of the glomerulus and the Bowman capsule around it being clear and undamaged. In addition, the kidney tubular structure in the healthy rats shows that the structure is still intact with a marked large lumen and a cube-shaped epithelium in layers. The normal glomerulus is characterized by complete structure and intact Bowman capsules. Tubular epithelial cells appeared to be normal with a layer of epithelial cube layers and are arranged with clearly visible cell nuclei.

Dramatic changes occurred in the histopathological features of the kidney glomerular and tubular in the diabetic group (Figure 2(b)). The morphologic lesions of the glomerulus have been shown with a narrowed capsule Bowman structure, glomerular necrosis, and hypertrophy. Hypertrophy is an increase in the volume of organs or tissues due to an enlargement of cell components. The obstruction of the proximal tubular was shown in the proximal tubules.

There were repair and improvements in the histopathological profiles in the rat groups 3, 4, and 5, as shown in Figures 2(c), 2(d), and 2(e). In the treatment group with 250 mg/kg body weight (Figure 2(c)), the Bowman capsules and glomerulus sizes were narrowed down and tubules epithelium also showing improvement. In groups 4 and 5, repair in the kidney histopathological profiles was shown; however, hypertrophy in the glomerulus and proximal tubules still appeared, as glomerulus sizes did not return to normal, and the Bowman capsule was not narrowed down.

## 4. Discussion

Diabetic animal models that were created by the administration of streptozotocin are typically used as an animal disease model for diabetes research [13]. Hyperglycemia due to streptozotocin induction increased the production of free radicals, mostly nitric oxide radicals. Accumulation of excessive free triggers oxidative stress that causes disrupt in pancreatic beta cells, leading to DNA fragmentation of these cells. These cause inhibition of insulin synthesis and secretion, and as a result, insulin levels decrease and blood glucose sugar levels increase [11].

In the present study, treatments with 250, 375, and 500 mg/kg body weight of hydroethanolic root extracts of *R. tuberosa L* caused a significant decrease in kidney MDA levels. The decrease in MDA levels in the treatment groups can occur due to antioxidant compounds in the *R. tuberosa* L. Our previous study showed that the hydroethanolic extracts of *R. tuberosa L* contained sorbifolin, cirsimaritin, cirsimarin, and cirsiliol-4-glucoside, which were flavonoid compounds [22]. Flavonoids have been identified to have high antioxidant activity [24–26]. The mechanism of flavonoids as antioxidants is conducted directly by donating hydrogen ions; as a result, they neutralize the effects of free radicals [27, 28]. Moreover, the indirect mechanism of flavonoids is to increase the expression of endogenous anztioxidant genes through several mechanisms, one of which is the activation of nuclear factor erythroid 2 related factor 2 (Nrf2), which is a gene that plays a role in the synthesis of endogenous antioxidant enzymes [29]. Increased levels of antioxidants in the body can indirectly reduce oxidative stress; hence, lipid peroxidation reactions decrease and lead to decrease in MDA levels [30].

It also has been shown that, in a diabetic state, an imbalance of oxidant/antioxidant indicates a status of chronic inflammation. In this current study, diabetic rats experienced high levels of TNF alpha expression. These findings were in agreement with a previous study which reported that levels of TNF in the diabetic rats were prominently increased [31]. Decrease in the TNF alpha expression occurred in all three treatment groups. This is caused by the administration of therapy using *R. tuberosa L* root extract that reduced ROS activity. The ability of the extracts to reduce inflammation in the kidney could either be due to direct or indirect effects through reduced levels in hyperglycemia and oxidative stress. Flavonoid compounds in the *R. tuberosa L* root extract worked as an anti-inflammatory agent by suppressing proinflammatory cytokine expression, suppressing the production of ROS. Flavonoids can block several proinflammatory cytokines including TNF-*α*, IL-1*β*, and IL-6 [32]. Flavonoids also inhibit the activation of NF-K*β* due to the high rate of ROS; decreasing NF-K*β* activity causes a decrease in TNF alpha expression [33]. The decrease in NF-K*β* stimulates cell regeneration in necrotic tubules; therefore, TNF alpha expression in the kidney tubules decreases.

Diabetic nephropathy is the leading cause of chronic kidney disease. Several factors contribute in the development and progression of diabetic nephropathy including hyperglycemia, obesity, hypertension, smoking, hereditary, and advanced age [34]. In recent years, the role of inflammation and oxidative stress has been emphasized. Oxidative stress can increase the production of inflammatory cytokines, and an increase in inflammatory cytokines can stimulate oxidative stress. The MDA and TNF alpha are well correlated with oxidative stress and inflammation [34, 35]. Nonetheless, there are other targeting markers of inflammation (proinflammatory cytokines, adhesion molecules, and chemokines) and markers of oxidative stress (nitrotyrosine, isoprostanes, and S-glutathionylation family) that can be useful [34].

Degradation of kidney functions as observed in diabetic rats was also shown with the histopathological changes in the kidneys as indicated by atrophy of glomerular and reduction in surface area of the Bowman capsules. Following administration of hydroethanolic root extracts of *R. tuberosa L.*, interglomerular size variations were reduced, and glomerular diameter was increased. In addition, glomerular cross-sectional area and glomerular volume in diabetic rats were an indication of amelioration of renal hypertrophy resulting from treatment with the extract. In this study, capacity of extracts to some extent improving damage of renal function could be due to alleviation of oxidative stress, inflammation, apoptosis, and fibrosis in the kidney and enhancement of the kidney cell proliferation. These results were in agreement in results shown in the decreased levels of MDA and TNF alpha expression.

It is interesting to note that results in the MDA level, TNF alpha expression, and kidney histology profiles after administration of the treatments were not in a dose-dependent manner. Nonetheless, the lowest dose used (250 mg/kg body weight) in this study resulted in the most optimum effects. These can be explained that, in a case when flavonoids levels are high, flavonoids can change their function from antioxidants into prooxidants, causing a loss in hydrogen atom and resulting in increasing free radicals in the body, leading to increase in cell damage. The interplay of antioxidative and prooxidative role of antioxidants has been observed before [36]. A good example is a hydrophilic antioxidant, ascorbic acid (vitamin C). Ascorbic acid has the privilege to have antioxidant and prooxidant effects, depending upon the dose [37]. Furthermore, studies also indicated that natural antioxidants can act as prooxidants, which produce free radicals and cause DNA damage and mutagenesis. The prooxidant activity is typically catalyzed by transition metals like Fe and Cu that are generally present in the biological systems [38].

Toxicity of free radicals contributes to proteins and DNA injury, tissue damage, inflammation, and eventually leads to apoptosis [10]. Maintaining intracellular reactive oxygen species (ROS) content at low physiological concentration is vital for the survival of cells; hence, imbalanced metabolism and excess ROS production result in a range of disorders such as diabetes mellitus, neurodegenerative diseases, atherosclerosis, and rheumatoid arthritis [35]. Considering the above mentioned, high levels of antioxidants and scavenging agents in hydroethanolic root extract of *R. tuberosa L*. engage in reversing the toxic effect of oxidative stress in the cells. However, the exact molecular and cellular mechanisms in which the extracts can reverse the cytotoxicity of ROS remain unclear. Further molecular and cellular approaches are needed to elaborate into the mechanisms behind this event.

## 5. Conclusions

In conclusion, *R. tuberosa L* hydroethanolic root extracts have beneficial results on diabetes and its accompanying complications, in particular in diabetic nephropathy, through a significant decrease in the levels of kidney MDA and the TNF alpha expression. In addition, the effects have also been observed in the repair of kidney histopathological profiles. However, further molecular and cellular approaches are needed to underlie mechanisms of these positive effects, and the particular compounds facilitating the effects remain to be established.

## Figures and Tables

**Table 1:** MDA levels on the rats' kidney.

Group	MDA levels (*μ*g/mL)^*∗*^	Increasing or decreasing percentage
1	1.07 ± 0.21^a^	
2	4.08 ± 0.10^e^	281.31 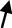
3	1.93 ± 0.18^b^	52.70 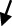
4	2.41 ± 0.11^c^	40.93 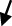
5	2.85 ± 0.14^d^	30.15 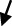

^∗^Different notations indicate significant differences between treatments for MDA levels (*p* < 0.05).

**Table 2:** TNF alpha expression on the rats' kidney.

Group	TNF alpha expression^*∗*^	Increasing or decreasing percentage
1	18.87 ± 4.85^a^	
2	53.65 ± 14.12^e^	185.23 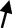
3	31.47 ± 4.90^b^	42.24 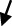
4	34.70 ± 3.20^c^	35.51 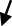
5	42.92 ± 10.53^d^	20.12 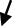

^∗^Different notations indicate significant differences between treatments for TNF-*α* expression (*p* < 0.05).

## Data Availability

The data used to support this study are included in the article and will be made available upon request.
